# Supplementary figures and images for: Commensal E. coli limits Salmonella gut invasion during inflammation by producing toxin-bound siderophores in a tonB-dependent manner
Source: PLoS Biol. 2024 Jun 12;22(6):e3002616. doi: 10.1371/journal.pbio.3002616 (PMC11168627; doi:10.1371/journal.pbio.3002616)

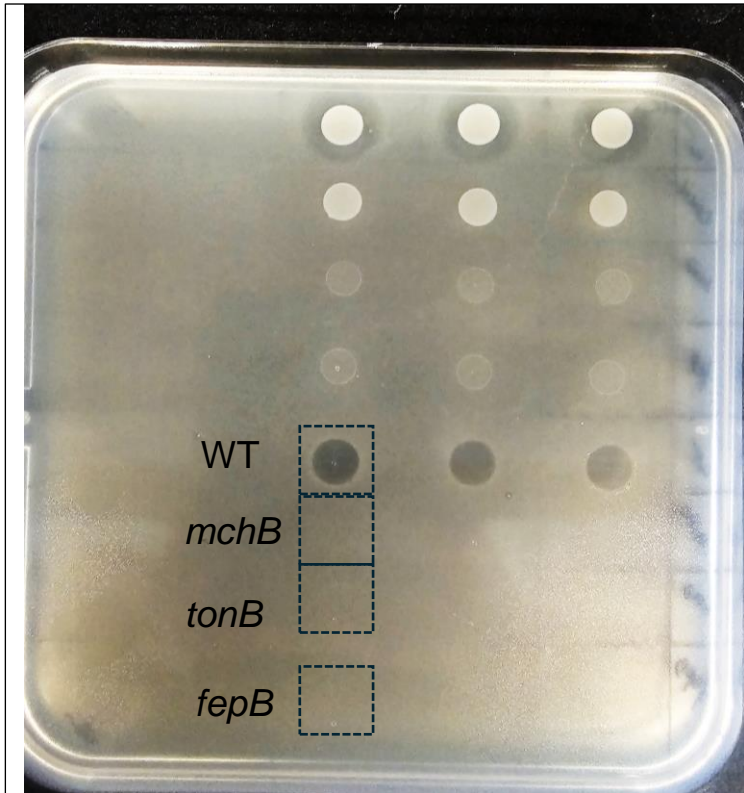

*E. coli* 8178 supernatant

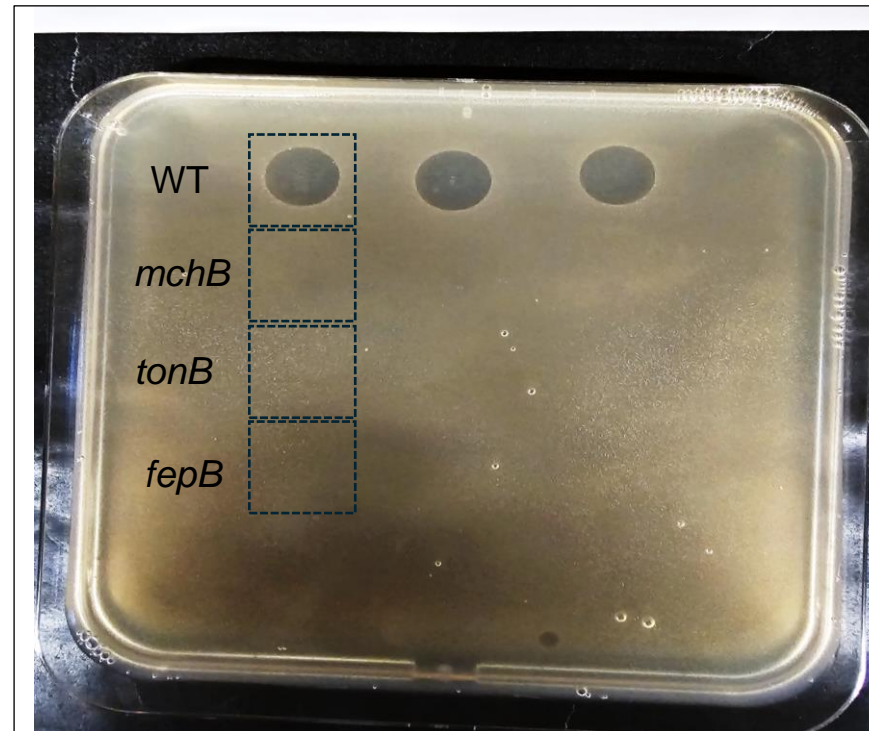

*E. coli* 8178 cytoplasmic content

Supplement: S1 Raw Images — (PDF) [file pbio.3002616.s009.pdf]
