# Supplementary material for: Commensal E. coli limits Salmonella gut invasion during inflammation by producing toxin-bound siderophores in a tonB-dependent manner
Source: PLoS Biol. 2024 Jun 12;22(6):e3002616. doi: 10.1371/journal.pbio.3002616 (PMC11168627; doi:10.1371/journal.pbio.3002616)
Supplement: Attachment — Submitted filename: Point-by-point rebuttal.pdf [file pbio.3002616.s011.pdf]

Reviewer #1:

A great study. Beautiful figures. I just have issues with the narrative and lack of contextualization with existing work. I hope you agree some changes along these lines might allow this work to be more useful to the field.

General comments:

I find the experimental design, hypothesis generating and testing, and conveying of results through figures to be really superb. It is a genuinely well thought-out and organized study. That said, I find that many of the results presented have previously been demonstrated in the literature, or are obvious implications of existing literature, without proper acknowledgement of that fact by the authors.

The manuscript largely focuses on the strain E. coli 8178, that appears to inhibit Salmonella via the same mechanisms already reported in the much more widely studied E. coli Nissle 1917 strain. There are many nuanced differences between the present study and the existing literature that a specialist in this area might be very interested to read. However, these differences are not discussed.

I trust the authors know the existing literature well, and I think a re-write of some of the narrative needs to give proper credit to past work, and better situate the current findings in light of published works. The final section of results (nor the discussion) not contextualizing the outcomes in the framework of Sassone-Corsi et al 2016 Nature, is a serious shortcoming. The authors do cite this paper and discuss it very briefly in the discussion, but the fact that these results are not really contextualized in the light of that study is very confusing. I think it would be much more beneficial to the scientific community if this work were to make an effort to demonstrate how it builds on previous research in this area, rather than largely ignore it. I know there are plenty of differences in the experimental design between Sassone-Corsi and the study at hand. Method of inducing inflammation a key difference between them. But this kind of contextualization which would be useful for the field is completely absent.

The finding that tonB and fepB mutations result in a loss of detectable microcin production in the assay performed here is indeed an interesting and perhaps unexpected result. I don't know if this phenomenon has previously been reported in the literature. However, this result is left as mostly an aside and not explored in much detail.

We would like to thank referee#1 for dedicating her/his time to review our manuscript. We are pleased to note the genuine interest expressed about our study and apologize for the lack of contextualization. We hope that our revised manuscript and detailed point-by-point responses will be compelling enough to support the publication of our work.

Major Comments

1) Line 147 - The validation of the method described seems to rely exclusively on the Benjamin et al paper which is in revision. Either the methods used for this aspect of the work should be described and validated in greater detail, or this manuscript should be published only once the Benjamin paper passes peer review. Additionally, are these targeted mutations or random? If targeted, how were these sites for mutagenesis selected?

<u>Response</u>: We agree with referee#1 that this section is not sufficiently described and apologize for that. Briefly, multiples 40bp-barcoded sequences were created and individually inserted into a fitness-neutral region of the *E. coli* 8178 genome (targeted mutation within a cryptic gene). The fitness of the barcoded *E. coli* 8178 strains was assessed *in vivo*, over a 4-day infection, and revealed no defect associated with the presence of the DNA tag. The strain construction and *in vivo* validation data will be published in the *Daniel et al* manuscript (accepted in *Nature Microbiology*: DOI 10.1038/s41564-024-01634-9) prior to our work. For the convenience of the readers, we have added additional information on our manuscript and have replaced:

The fitness of each individual mutant was assessed by quantitative PCR (qPCR) through the detection of a unique DNA barcode and calculated as a normalized competitive index (C.I)

by:

[L151] *The fitness of each individual mutant was assessed by quantitative PCR (qPCR) through the detection of a unique fitness-neutral 40bp-DNA barcode and calculated as a normalized competitive index (C.I)*

And added in the Method section:

[L400] *Gene deletion was confirmed by colony PCR. In a similar fashion, barcoded strains were created through the insertion of 40 bp-DNA WISH (Wild-type Isogenic Standardized Hybrid) tags within a fitness-neutral region of the E. coli 8178's genome (Daniel et al., 2024).*

2) Results Lines 168 - 215: The role of TonB in the uptake of both siderophores and siderophore-iron complexes is extremely well-established. This section seems written without that fact in mind. While Deriu 2013 is cited at the start, the authors seem to suggest that the results of that work only demonstrate that uptake of siderophores led EcN's ability to effectively compete with S. Tm. They then state tonB has a pleiotropic effect regarding siderophore iron complexes (again, the connection is extremely well-established). Deriu 2013 clearly state that their results suggest that it is the competition for iron (via siderophore-iron complexes) which leads to EcN-mediated competition with S. Tm. I find that this results section is written as though that is not a clear implication of Deriu 2013's data. All of the data provided are completely valid and clearly directly in line with the results of Deriu 2013's data. I don't understand why it is not written as such.
Additionally, the focus on FepB is confusing. The essential role of FepB in catecholate siderophore uptake (and catecholate siderophore-iron complexes) is clearly established. This all again confirms clear expectations for existing literature. To suggest Ec 8178 specifically relies on FepB to compete with S. Tm just confuses the larger point, that your data is further confirmation that 8178 competes with S. Tm for iron via catecholate siderophores, which is well-established.
The authors have great experiments and very clear data and really nice figures. I just think the narrative of this results section is currently written in a way that mostly ignores what is known and expected based on existing literature, which has the potential to confuse readers.

Response: We apologize for the lack of clarity in that section and thank referee#1 for giving us the opportunity to provide further clarification. While we acknowledge the well-established function of *tonB* and *fepB* in the uptake of siderophore-iron complexes, we initially chose to begin this section with the Deriu 2013 paper as it is one of the only studies highlighting the contribution of *tonB* in mediating *S*. Tm elimination by a commensal *E. coli*.

In the Deriu paper, the authors have nicely demonstrated that *E. coli* Nissle relies on the *tonB* gene to outcompete *S*. Tm. They further proposed a model where *E. coli* Nissle prevents *S*. Tm growth through a *tonB*-dependent iron limitation. Consistent with this work, we observed that an *E. coli* 8178 *tonB* mutant has lost its ability to outcompete *S*. Tm. We acknowledge referee#1's observation that this aligns nicely with the Deriu 2013 work, suggesting that *E. coli* 8178 outcompetes *S*. Tm through a mechanism involving *tonB*-dependent iron-siderophore uptake systems. However, we intentionally chose not to assert such claim. Notably, apart from the well-established pleiotropic function of *tonB*, it remains largely unclear from a mechanistic standpoint how *E. coli* 8178 (and ultimately *E. coli* Nissle) could prevent the growth of *S*. Tm through catecholate siderophores while both strains share the same siderophore synthesis and uptake systems. Moreover, the utilization of catecholate siderophores as a carrier for toxin delivery (Sassone-Corsi et al., 2016) indicates that these molecules are commonly exchanged between the *E. coli* strains that produce them and their niche competitors (here *S*. Tm). This contradicts the idea that *E. coli* 8178 (and *E. coli* Nissle) monopolize catecholate siderophores at the expense of *S*. Tm growth. Consequently, and in the absence of further validations, we refrained from drawing any conclusions about catecholate intake at that stage. Instead, we opted to further investigate the effect of *tonB* mutation on *E. coli* 8178's competitiveness. This follow-up investigation ultimately revealed that the loss of competitiveness in a *tonB* mutant was attributed to its incapacity in producing microcins rather than its inability to uptake catecholate siderophores, which differs from what was drawn from the Deriu 2013 manuscript.

We have revised the corresponding section by (1) mentioning the well-established function of *tonB* prior to introducing the Deriu work, (2) removing the confusion around the pleiotropic effect of *tonB* and (3) presenting our results in the context of the Deriu 2013 manuscript. We are now clearly highlighting the overlapping findings with *E. coli* Nissle, without formally stating the iron deprivation hypothesis which turned out to be not applicable in our conditions.

Specifically, we have replaced:

Similar to E. coli 8178, recent work in the literature demonstrated the contribution of the E. coli Nissle tonB gene in outgrowing S. Tm in the gut (Deriu et al., 2013). This was attributed to the incapacity of an E. coli Nissle tonB mutant to uptake siderophores, thereby allowing the bloom of S. Tm in the inflamed gut. However, it is worth noting that tonB has a pleiotropic function and less is known about the detailed mechanism by which it affects E. coli competitiveness against S. Tm. TonB is an inner membrane protein involved in the uptake of vital metabolites such as vitamin B12 and iron-siderophore complexes (Noinaj et al., 2010). To identify the specific tonB-related pathway responsible for S. Tm elimination, we tested a set of different tonB-dependent receptor (TBDR) mutants in E. coli 8178.

by:

[L177] *TonB is an inner membrane protein facilitating the energy-dependent transport of several molecules (Wookey, 1982; Klebba et al., 1993). Among these are vitamin B12 and iron-siderophore complexes, which were shown to transit through different tonB-dependent receptors (TBDR) (Noinaj et al., 2010). Notably, recent work in the literature demonstrated the contribution of the E. coli Nissle tonB gene in limiting S. Tm growth in vivo (Deriu et al., 2013). This effect was attributed to the incapacity of an E. coli Nissle tonB mutant to compete for iron, thereby allowing the bloom of S. Tm in the inflamed gut (Deriu et al., 2013). Drawing from this example, we speculated that E. coli 8178 outgrows S. Tm through iron acquisition. To test this hypothesis and identify the specific tonB-related pathway responsible for S. Tm elimination, we tested a set of different TBDR mutants in E. coli 8178.*

and:

We thus concluded that E. coli 8178 relies on fepB, specifically through catecholate siderophore intake, to outcompete S. Tm in the inflamed gut

by:

[L212] *We thus concluded that, similar to E. coli Nissle, E. coli 8178 relies on catecholate siderophore to outcompete S. Tm in the inflamed gut (Deriu et al., 2013).*

3) Line 239 - Many strains which harbour the Class IIB microcin production cluster also encode for a homolog of IroB, which is frequently identified as MchA. It glycosylated enterobactin into salmochelin, just as IroB does. Does 8178 have MchA? Without knowing this information, I cannot assess whether or not the claim of Line 239 is reasonable or not. If it is true that the iroB deletion mutant cannot make salmochelin, the claim should still be amended to state that 'attenuation of S. Tm by E. coli 8178 is solely dependent on microcin H47 synthesis rather than salmochelin acquisition.' You could also confirm what siderophores are present experimentally, using biochemical methods rather than relying on genetics.

Response: This is a very valid question, and we thank referee#1 for pointing this out. We could not find any homologous gene of *mchA*, indicating that *E. coli* 8178 relies solely on *iroB* to convert enterobactin into salmochelin. From that, we can confirm that a *mchB*/*iroB* double mutant is incapable of producing microcin H47 and salmochelin siderophore. Notably, we observed that the competitiveness of this strain against *S*. Tm is comparable to a microcin mutant (single *mchB* knock out, Fig. 4B). This suggested that, despite the apparent overlap with the Deriu work, *E. coli* 8178 relies on microcin, rather than salmochelin acquisition, to outcompete *S*. Tm.

We have added the missing information on *mchA* and modified the result as requested. Specifically, we have added replaced:

A similar trend was observed with an iroB mutant strain which is incapable of converting enterobactin into salmochelin (Figure 3D; Supplementary Fig. 3E).

by:

[L223]. *A similar trend was observed with an iroB mutant strain which, lacking its homolog mchA, is incapable of converting enterobactin into salmochelin (Fig 3D; S3E).*

and changed:

Notably, an E. coli 8178 deficient for both microcin H47 and salmochelin synthesis (mchB/iroB) phenocopies a mchB mutant, confirming that the growth attenuation of S. Tm by E. coli 8178 is solely dependent on microcin H47 synthesis rather than iron acquisition.

by:

[L251] *Notably, an E. coli 8178 deficient for (1) microcin H47 and salmochelin synthesis (mchB/iroB) or (2) microcin H47 synthesis and salmochelin intake (mchB/iroN), phenocopies a mchB mutant (Fig 4B, S4A). This confirmed that that the growth attenuation of S. Tm by E. coli 8178 is solely dependent on microcin H47 synthesis rather than salmochelin acquisition.*

We also agree with referee#1 that our conclusions rely mainly on genetic approaches. While we acknowledge that biochemical assays could have nicely complemented our study, it is important to note that most of the biochemical work in the literature reporting the isolation/detection of microcins and/or siderophores in *E. coli*/S. Tm were done by mass spectrometry on controlled and defined media (Vassiliadis et al., 2010; Henderson et al., 2009; Behnsen et al., 2021). Applying these approaches in an *in vivo* context poses significant challenges, particularly due to the complexity and heterogeneity of gut luminal samples. For that reason and given the well-established literature on catecholate siderophore synthesis and uptake in *Enterobacteriacea* (Mey et al., 2021), we capitalized on the existing body of literature to shape our study framework.

We mentioned the current limits in detecting/isolating siderophores/microcins from *in vivo* samples in the results. We substituted.

To further demonstrate E. coli 8178's ability to produce microcin H47 in the inflamed gut, we designed an E. coli 8178 mchI/mchB double mutant which lacks the gene responsible for microcin immunity and synthesis, making it susceptible to microcin-dependent killing by the WT strain.

by:

[L261] *To further prove that inflammation acts as a cue to trigger microcin H47-dependent competition, and considering the difficulties associated with detecting microcin H47 from in vivo samples, we designed an* E. coli *8178 mchI/mchB double mutant.*

4) Results Line 216 - 266: The lack of reference to Sassone-Corsi et al Nature 2016 in this section is confusing. Again, these results seem to hold very well in light of that work. Granted, it seems here the effect is largely due to Microcin H47, rather than microcin M, in regards to the inhibitory effect against a strain of S. Tm. Though, in vitro tests have demonstrated the inhibitory potential of microcin H47 against strains of S. Tm (Palmer et al, 2020).

Response: We agree with the referee#1 that the Sassone-Corsi 2016 work reference is missing in this section. We have now mentioned it and highlighted the key differences compared to our study. Specifically, we have added:

[L240] *This is further illustrated by E. coli Nissle, which was shown to eliminate S. Tm through the secretion of siderophore-bound microcins in vivo (Sassone-Corsi et al., 2016).*

Additionally, we have substituted:

Furthermore, in trans expression of the mchI immunity gene in S. Tm increased the pathogen's survival against E. coli 8178 (Figure 4D) which all together indicate that E. coli 8178 produces microcin-H47 to eliminate S. Tm in vivo.

by:

[L267] *Finally, in trans expression of the mchI immunity gene in S. Tm increased the pathogen's survival against E. coli 8178 (Fig 4E; S4C). Taken together, these data indicate that in contrast to E. coli Nissle that predominantly uses microcin M (Sasonne-Corsi et al., 2016), E. coli 8178 produces microcin H47 in an inflammation-dependent manner to eliminate closely related competitors such as S. Tm in vivo.*

We are also now referring to the published work by Palmer et al, demonstrating the *in vitro* activity of microcin H47 in the Discussion section. Specifically, we have added:

[L338] *As a result, microcin H47 was shown to exert an antibacterial activity against several members of the Enterobacteriaceae family) in vitro (Palmer et al., 2020; Mortzfeld et al., 2022), a finding now corroborated in vivo, using the natural mouse commensal isolate E. coli 8178.*

5) Comparing figure 3A and 4D, if the primary cause of S. Tm inhibition is indeed due to Microcin H47, then I would expect a 8178 tonB mutant and a mchB mutant to result in similar levels of S. Tm (based on your experiments showing that TonB mutants are not producing microcins). However these seem to have roughly a 10-fold difference, where S. Tm in competition with an 8178 tonB mutant are doing ~10x better than compared to competition with a mchB mutant. How do you account for these differences? It seems to imply there is likely competition over both consuming iron and via microcins, which is not reflected in the interpretation of results.

Response: We thank referee#1 for highlighting the differing levels of *S*. Tm competition associated with the *E. coli* 8178 *tonB* and *E. coli mchB* strains.

Throughout our study, we found that *E. coli* 8178 relies on microcin H47 to outcompete *S*. Tm in the gut. However, it is worth mentioning that the level of *S*. Tm survival depicted in presence of a microcin H47-deficient *E. coli* strain is consistently lower compared to *S*. Tm mono-infected mice (Fig.4B). This indicated to us that, aside from microcin H47, *E. coli* 8178 likely employs additional factors restricting the growth of *S*. Tm. We are actively investigating this aspect further.

When comparing Fig. 2C and Fig.4B, we can observe that the *E. coli tonB* mutant (which cannot uptake siderophores and produce microcin H47) is even less effective at outcompeting *S*. Tm than the *E. coli mchB* strain (which cannot produce microcins). While this suggests that *E. coli* 8178 may also use siderophore acquisition to outcompete *S*. Tm, it's important to interpret these data cautiously. Indeed, upon co-infecting mice with *S*. Tm and the *E. coli tonB* mutant, we found that this latter experienced a significant fitness defect *in vivo* (Supplementary Figure 2B), which we already stated in the text as follow:

[L171] *However, this attenuated competitive effect was associated with a reduced E. coli 8178 tonB gut colonization, raising the alternative hypothesis that tonB deletion could indirectly affect E. coli 8178-mediated competition by impacting bacterial growth.*

Due to the disparate level of gut colonization between the *E. coli* 8178 *tonB* and *mchB* mutants, we believe that making such assumptions, without any further validations, remains extremely speculative.

Besides the *E. coli* 8178 *tonB* mutant, we would like to bring to the referee's attention that *E. coli* 8178 *fepB* strain was also extremely inefficient in outgrowing *S*. Tm (Figure 3C), with the recovered *S*. Tm load being higher than in the presence of a *mchB* mutant (Figure 4B). Although the *E. coli* 8178 *fepB* did not experience any gut colonization defect in these conditions (Supplementary Figure 3D), we refrain from making conclusion about catecholate siderophore acquisition aiding in outcompeting S. Tm. As mentioned above, catecholate siderophores, and particularly salmochelin (known to be the main catecholate siderophore supporting *S*. Tm growth in the inflamed gut: Fischbach et al., 2006; Rafatellu et al., 2009), can serve as carriers for the delivery of toxic molecules (microcin M, microcin H47), contradicting with the principle of privatizing siderophores. In line with this, we found that a *mchB/iroB E. coli* 8178 double mutant (incapable of producing microcin H47 and salmochelin) phenocopied the single *mchB* knockout strain (Figure 4B), hence refuting the hypothesis of an

additional mechanism relying on iron acquisition. To reinforce this argument, we have now created an *E. coli* 8178 strain unable to synthetize microcin H47 and uptake salmochelin siderophore. For that purpose, we targeted the *iroN* receptor gene which was previously shown to mediate salmochelin uptake in *S*. Tm (Hantke et al., 2003). Notably, the *E. coli* 8178 *mchB/iroN* strain phenocopied the *mchB* mutant, which further proves that salmochelin uptake does not play any role in the *E. coli* 8178- *S*. Tm competition. Based on that, we believe that the increased *S*. Tm survival observed in presence of the *fepB* mutant is not attributed to iron acquisition deficiency but may account from other factors associated with its pleiotropic function.

We have updated Figure 4B and Supplementary Figure 4A by adding the *in vivo* competitiveness of the *E. coli* 8178 *mchB/iroN* against *S*. Tm. We have revised the text accordingly and replaced:

Notably, an E. coli 8178 deficient for both microcin H47 and salmochelin synthesis (mchB/iroB) phenocopies a mchB mutant, confirming that the growth attenuation of S. Tm by E. coli 8178 is solely dependent on microcin H47 synthesis rather than iron acquisition (Figure 4B, Supplementary Figure 4A).

by:

[L251] *Notably, an E. coli 8178 deficient for (1) microcin H47 and salmochelin synthesis (mchB/iroB) or (2) microcin H47 synthesis and salmochelin intake (mchB/iroN), phenocopies a mchB mutant (Fig 4B, S4A). This confirmed that that the growth attenuation of S. Tm by E. coli 8178 is solely dependent on microcin H47 synthesis rather than salmochelin acquisition.*

We have additionally updated the discussion and changed:

*This observation, coupled with the redundant effect of a microcin H47- and siderophore synthesis-deficient strain (mchB/iroB), suggested that the lost competitiveness of the E. coli 8178 tonB or fepB mutant against S. Tm is not attributed to a defect in iron acquisition but rather to the incapacity of producing microcin H47 (**Fig 4B, E**).*

by:

[L362] *This observation, coupled with the redundant effect of a microcin H47- and siderophore synthesis-deficient strain (mchB/iroB), or a microcin H47-synthesis and salmochelin uptake-deficient mutant (mchB/iroN), suggested that the lost competitiveness of the E. coli 8178 tonB or fepB mutant against S. Tm is not attributed to a defect in iron acquisition but rather to the incapacity of producing microcin H47 (**Fig 4B,F,G**).*

6) Figure 4 has me somewhat confused. 4A, 4B, and 4D all have the same X and Y axis and the same experimental set-up, but the data-points are clearly different. Taking the 8178 mchB mutants for example, why aren't all the data points pooled, or used across the different comparisons that you wish to show? Perhaps this is a nuance of high-throughput animal work that I'm unfamiliar with. It seems like an unfortunate waste of mice to be essentially repeating the same experiment over and over (e.g. S.Tm alone, S.Tm vs 8178 WT; S.Tm vs 8178 mchB_KO) for each plot subpanel.

Response: Figure 4A, 4B and 4E (formerly 4C in the old version) represent data from several independent experiments. Although colonization levels of the *S*. Tm alone, *S*. Tm + *E. coli* 8178 and *S*. Tm + *E. coli* mchB conditions remained consistent (~10^9, 10^6, 10^8 respectively), we are unfortunately unable to use the same dataset for different figures. Indeed, despite our standardization efforts, the outcome of mouse intestinal infections remains multifactorial and variable. For this reason, and to ensure the reliability and accurate interpretation of our results, full controls are advisable for each independent experiment.

minor comments

7) The abstract seems to imply that the OM receptors of Microcin H47 being TonB-dependent catecholate receptors was unappreciated, though this is well established in the literature. Clear experiments demonstrate this in Patzer et al 2003 - https://doi.org/10.1099/mic.0.26396-0.

Response: We agree with the referee that the uptake of microcins by TonB-dependent catecholate receptors is widely established in the literature. However, by claiming the following statement: *This protective function was compromised upon disruption of the tonB-dependent catecholate siderophore uptake system, highlighting a previously unappreciated crosstalk between iron intake and microcin H47 activity*, we are mentioning the loss of microcin activity upon disruption of *tonB* and *fepB* gene in the microcin-producing *E. coli* 8178 strain. As far as we are aware, such crosstalk has not been demonstrated.

To clarify this, we have now specified the strain in which this effect was visible, as follows:

[L24] *This protective function was compromised upon disruption of the E. coli 8178 tonB-dependent catecholate siderophore uptake system, highlighting a previously unappreciated crosstalk between iron intake and microcin H47 activity.*

8) Line 72 - Is the co-blooming necessarily due to strong metabolic overlap between these species? Is this demonstrated in the referenced literature for this sentence? Could it also simply be due to things like increase in oxygen?

Response: *S*. Tm-elicited inflammation is associated with alterations in the gut luminal condition. As mentioned by the referee, oxygen, which increases significantly during inflammation, promotes the growth of *S*. Tm and other facultative anaerobes, such as *E. coli*, in these conditions (reviewed in Byndloss et al., 2018). Alongside oxygen, inflammation is followed by the release of additional electron acceptors such as tetrathionate (Winter et al., 2010) and nitrate (Winter et al., 2013) which both *S*. Tm and *E. coli* can use to thrive. Given this shared metabolic capacity (with "metabolic" here not limited to carbohydrate utilization), it is commonly assumed that the metabolic overlap between these 2 species facilitate their co-proliferation during inflammation. Due to these considerations, we have chosen not to modify our text

9) Figure 1 - It is hard to see the black mean lines associated with the black data points. Perhaps consider using data points which are somewhat lighter (more gray) in order for the black line to be observable.

Response: We have changed the colour in all figures.

10) Line 111 - I don't understand the logic here. At 24 p.i., inflammation levels are reduced when co-infected, relative to S. Tm alone. Doesn't this imply some competition at 24hr p.i.? While it may not be the inflammation-induced competition mechanisms, it seems like it would indicate there is competition for nutrients, space, etc., reducing S. Tm's ability to cause inflammation.

Response: This is a highly pertinent observation, and we thank referee#1 for bringing this up. Despite inducing a significant competitive phenotype visible at 96 h post-infection, we found that the presence of *E. coli* 8178 has no impact on the *S*. Tm-elicited inflammation at this stage of infection (the lipocalin levels are similar between *S*. Tm alone and S. Tm + *E. coli* 8178 conditions at 96 h post-infection – Figure 1C). It's worth noting that microcin H47-mediated *S*. Tm killing is initiated only when gut inflammation is established, which occurs too late for *E. coli* 8178 to mitigate it.

A noticeable disparity in the lipocalin-2 levels, however, becomes apparent at 24 h post infection. This difference nicely correlates with the slight decrease in *S*. Tm load visible in presence of *E. coli* 8178 at 24 h post-infection (Figure 1B), indicating of a competition occurring at that stage. As we are currently investigating it, we have reasons to believe that this early competition (occurs only at 24 h post-infection) is an independent event not reliant on microcin H47, but rather driven by nutrient acquisition. Given our limited understanding of this phenomenon at that stage, we chose not to further interpret this phenotype. Instead, we directed our focus toward the more pronounced competition observed at 96 h post-infection, which we highlighted in the text as follow:

[L103] *Besides the slight decrease (~ 10-fold) in S. Tm loads visible at the early stage of infection (24 h p.i), co-infection with a 1:1 ratio of S. Tm and E. coli 8178 led to equivalently high gut pathogen luminal loads after 48 h of infection (Fig 1B and S1A). This load displayed a more striking decline (1.000- to 10.000-fold) 72 to 96 h post-*

infection (Fig 1B and S1A) which aligned with previous observation in C57BL/6 mice (Stecher et al., 2012) and prompted us to investigate the underlying mechanisms.

11) Figure 2A - The fitness plots are unnecessary here. Either denote clearly in the caption that this is mock data for the purpose of demonstrate, or exclude this aspect of the experimental schematic.

Response: We have now clarified in the caption that the graph presents mock data.

12) Line 171 -To say that tonB has a pleiotropic function where, not only is it involved in siderophore uptake, but also the uptake of siderophore-iron complexes seems a bit misleading. This function of TonB is well-established.

Response: As mentioned above, we revised this section by highlighting the overlapping findings with the Deriu 2013 and removing any reference to the pleiotropic function of *tonB*.

13) Line 248 - From your own experiment, as well as experiments from Sassone-Corsi at al 2016, E. coli can clearly use these microcins to kill other E. coli as well. So it seems inappropriate to state that 8178 produces H47 to kill S. Tm. In the experimental design where S. Tm is present, yes that is agreed upon, but it seems to target any susceptible species present under inflammation conditions. While Salmonella is a common cause of inflammation, it is by no means the only.

Response: We agree with referee#1 that microcin H47 are not meant to exclusively outcompete *S.* Tm but rather directed toward any susceptible species able to uptake catecholate siderophores. We have clarified this point and specifically replaced:

*Furthermore, in trans expression of the mchI immunity gene in S. Tm increased the pathogen's survival against E. coli 8178 (**Figure 4D**) which all together indicate that E. coli 8178 produces microcin-H47 to eliminate S. Tm in vivo.*

by:

[L269] *Taken together, these data indicate that in contrast to E. coli Nissle that predominantly uses microcin M (Sasonne-Corsi et al., 2016), E. coli 8178 produces microcin-H47 to eliminate closely related competitors such as S. Tm in vivo.*

14) Line 287 -Indicate the type of filter used for the supernatant assays. Also indicate the volume of filtered supernatant deposited onto the agar.

Response: The missing information have now been added in the Method section. Specifically, we have replaced:

The presence of microcin H47 was monitored by incubating the filtered (0.2 µm) supernatant of the attacker strains onto the LB agar prey mix, at 37 ºC overnight.

by:

[L452] *The presence of microcin H47 was monitored by incubating 5µL of the 0.2 µm-filtered supernatant from the attacker strains onto the LB agar containing the prey mix, overnight at 37 ºC.*

References:

- Behnsen, J., Zhi, H., Aron, A. T., Subramanian, V., Santus, W., Lee, M. H., ... & Raffatellu, M. (2021). Siderophore-mediated zinc acquisition enhances enterobacterial colonization of the inflamed gut. *Nature communications*, *12*(1), 7016.
- Byndloss, M. X., Pernitzsch, S. R., & Bäumler, A. J. (2018). Healthy hosts rule within: ecological forces shaping the gut microbiota. *Mucosal immunology*, *11*(5), 1299-1305.
- Fischbach, M. A., Lin, H., Zhou, L., Yu, Y., Abergel, R. J., Liu, D. R., ... & Smith, K. D. (2006). The pathogen-associated iroA gene cluster mediates bacterial evasion of lipocalin 2. *Proceedings of the National Academy of Sciences*, *103*(44), 16502-16507.

- Hantke, K., Nicholson, G., Rabsch, W., & Winkelmann, G. (2003). Salmochelins, siderophores of Salmonella enterica and uropathogenic Escherichia coli strains, are recognized by the outer membrane receptor IroN. *Proceedings of the National Academy of Sciences*, *100*(7), 3677-3682.
- Henderson, J. P., Crowley, J. R., Pinkner, J. S., Walker, J. N., Tsukayama, P., Stamm, W. E., ... & Hultgren, S. J. (2009). Quantitative metabolomics reveals an epigenetic blueprint for iron acquisition in uropathogenic Escherichia coli. *PLoS pathogens*, *5*(2), e1000305.
- Mey, A. R., Gómez-Garzón, C., & Payne, S. M. (2021). Iron transport and metabolism in Escherichia, Shigella, and Salmonella. *EcoSal plus*, *9*(2), eESP-0034.
- Palmer, J. D., Mortzfeld, B. M., Piattelli, E., Silby, M. W., McCormick, B. A., & Bucci, V. (2020). Microcin H47: A Class IIb microcin with potent activity against multidrug resistant Enterobacteriaceae. *ACS Infectious Diseases*, *6*(4), 672-679.
- Raffatellu, M., George, M. D., Akiyama, Y., Hornsby, M. J., Nuccio, S. P., Paixao, T. A., ... & Bäumler, A. J. (2009). Lipocalin-2 resistance confers an advantage to Salmonella enterica serotype Typhimurium for growth and survival in the inflamed intestine. *Cell host & microbe*, *5*(5), 476-486.
- Vassiliadis, G., Destoumieux-Garzón, D., Lombard, C., Rebuffat, S., & Peduzzi, J. (2010). Isolation and characterization of two members of the siderophore-microcin family, microcins M and H47. *Antimicrobial agents and chemotherapy*, *54*(1), 288-297.
- Winter, S. E., Thiennimitr, P., Winter, M. G., Butler, B. P., Huseby, D. L., Crawford, R. W., ... & Bäumler, A. J. (2010). Gut inflammation provides a respiratory electron acceptor for Salmonella. *Nature*, *467*(7314), 426-429.
- Winter, S. E., Winter, M. G., Xavier, M. N., Thiennimitr, P., Poon, V., Keestra, A. M., ... & Bäumler, A. J. (2013). Host-derived nitrate boosts growth of E. coli in the inflamed gut. *science*, *339*(6120), 708-711.

— — — — —

Reviewer #2 (Vanni Bucci):

In this paper, Cherrak et al. present a comprehensive exploration of the mechanisms underlying the colonization resistance exerted by commensal E. coli strain 8178 against Salmonella Typhimurium (STm). Through several in vivo experiments, the authors reveal that E. coli 8178, in the inflamed gut, outcompetes STm by producing microcin H47. Notably, this study in a notable advancement as, until now, microcin H47-mediated inhibition of STm had only been demonstrated through in vitro assays (Palmer et al., 2018; Palmer et al., 2020; Mortzfeld et al., 2022). The findings of this paper not only extend those of Sassone Corsi et al. (2016), who demonstrated H47-mediated inhibition in vivo of pathogenic E. coli but not Salmonella but also harmonize in vitro observations from recent years with in vivo experiments.

Response: We thank referee#2 for investing time reviewing our manuscript and for the encouraging comments on our work.

Two primary questions arise from this research:

1) Reconciliation with Sassone Corsi 2016 Results:
The paper prompts an inquiry into reconciling its results with Sassone Corsi 2016, particularly regarding the roles of microcin M and H47 in inducing lower levels of STm. In the Sassone Corsi study, knocking out mcmA and mchB independently suggested that microcin M, not H47, led to decreased STm levels. Given the apparent reversal in this paper's findings, have the authors considered potential reasons for this discrepancy? Additionally, recent work (https://www.biorxiv.org/content/10.1101/2023.12.05.570296v1) indicates that both

microcins are active against multiple strains of STm. How do the authors interpret these collective observations?

Response: We thank referee#2 for raising the differential contribution of microcin H47 and M in our study. Despite encoding both microcins, our *in vivo* data suggest that *E. coli* 8178 primarily relies on microcin H47 to outcompete *S*. Tm in the mouse gut. This observation contrasts with previous *in vivo* work demonstrating the contribution of microcin M (and not H47) against *S*. Tm (Sassone-Corsi et al., 2016), as well as recent *in vitro* data highlighting the activity of both microcins against multiple strains (Mortzfeld et al, biorxiv). At this stage, we lack a clear mechanistic understanding of these discrepancies. However, we believe that several factors may contribute to it.

1 - Regulation: Microcins M and H47, due to their functional similarities, are often encoded within the same operon, and thus subjected to the same regulation scheme. However, a closer look at the *E. coli* 8178's genome indicated the presence of an intergenic region between the microcin H47 synthesis/export cluster and the microcin M synthesis gene. Analysis of this specific region (using a promoter predictor software: Salamov et al., 2011) predicted the presence of an additional regulatory site (*rpoD*) that seems to be specifically present upstream of the microcin M synthesis gene (and not upstream the microcin H47 synthesis cluster). This may indicate a potentially different mode of regulation between both molecules. Besides regulatory proteins, non-coding RNA sequences have also been shown to regulate toxin expression (Jurėnas et al., 2022) which may further contribute to the hypothetical different expression of the microcin H47 and M clustered genes.

2- The strain specificity. In previous work, microcin M was shown to inhibit the growth of *S*. Tm *in vivo*, whereas microcin H47 appeared to have no influence (Sassone-Corsi, 2016). As the referee pointed out, our findings present contradictory conclusions. However, it is important to note that the *S*. Tm strain used in both studies is different (with ATCC 14028 in Sassone-Corsi et al 2016; and SL1344 in the current work), suggesting that the activity of microcin H47 and M depends on the target strain. In the Mortzfeld et al Biorxiv study, microcin M and H47 were shown to be both active against several *S*. Tm serovars (19585, 29630, BAA 190) that differ from the strains tested *in vivo* and thus poses challenges in reconciling data. Notably, *Proteus mirabilis* appears to display sensitivity toward a specific microcin (susceptible to microcin H47, resistant to microcin M) in this manuscript, which reinforces the idea of a microcin / target couple. In line with this, both microcin M and H47 seem active against *Klebsiella aerogenes*, while the closely related *Klebsiella oxytoca* is specifically inhibited by microcin M. From that, it's clear that microcin M and H47, despite sharing an apparently similar structure, exhibit a different target spectrum, which may account for the current discrepancies in the literature.

We have highlighted this aspect in the discussion section and added the following sentence:

[L343] *Besides the mouse commensal E. coli 8178 which relies on microcin H47, the human probiotic E. coli Nissle was shown to predominantly utilize a different class IIb microcin family member, microcin M, to mediate the elimination of S. Tm ATCC 14028 from the mammalian gut (Sassone-Corsi et al., 2016). Although the reasons why E. coli 8178 and Nissle utilize different microcins to target S. Tm serovars remain unclear, recent in vitro studies revealed a heterogenous target spectrum of class 2B microcins (Motzfeld et al., 2023). This suggests the existence of specific microcin/target pairings and hints at undiscovered resistance mechanisms that will be an interesting topic for future work.*

2) **Impact of tonB on H47-Mediated Killing:**

The paper introduces the novel insight that H47-mediated inhibition is dependent on the functionality of tonB in the producing strain. This prompts a question about the specific influence of tonB on H47-mediated killing. Considering that active molecules require the linkage of siderophores to immature microcin and subsequent export as mature microcin, does tonB impact siderophore production, potentially resulting in the secretion of only immature microcin (McHB)? Referring to Palmer 2020, which elucidates the relationship between siderophores and microcin activation, could tonB's role be integral to this process?

Response: This is an extremely relevant question, and we would like to thank the referee for pointing this out. While investigating the lack of competitiveness of the *E. coli* 8178 *tonB* (and *fepB*) mutant against *S*. Tm, we unexpectedly uncovered a connection between these proteins and microcin H47-dependent killing (Figure 4F). Initially, considering the recently discovered contribution of TonB-dependent receptors in protein secretion (Gómez-Santos et al., 2019), we hypothesized that *tonB* deletion might impact microcin H47 secretion. However,

our findings revealed that the cytoplasmic content of the *E. coli* 8178 *tonB* (and *fepB*) strain did not contain any detectable microcin H47 activity, suggesting that TonB (and FepB) may influence microcin H47 production instead.

Given the multistage synthesis process of microcin H47, which involves the production of the precursor, the siderophore, and their attachment, it remains unclear which specific step is affected by TonB (and FepB). To gain further insights, we have designed a microcin H47 reporter plasmid combining the promoter of the microcin synthesis cluster to a nanoluciferase gene. We have validated the reporter by demonstrating that microcin H47 expression is (1) triggered in our *in vivo S*. Tm infection model and (2) absent in non-inflamed condition (Figure 4C), which nicely correlates with the data presented in Figure 1D. Following its validation, we introduced the reporter plasmid into the *E. coli* 8178 *tonB* and *fepB* mutant and incubated the cells under iron-deprived condition. To our surprise, we found that deletion of *tonB* and *fepB* did not impact the expression of the microcin H47 synthesis cluster, indicating a potential link between *tonB* and siderophore synthesis and/or microcin maturation instead. The validation of the reporter plasmid has been added in Figure 4C. The microcin H47 reporter expression level in the *tonB* and *fepB* background is illustrated in Supplementary Figure 4C. We have revised the text accordingly and replaced:

Together, these data suggest that the attenuated S. Tm competition capacity of the E. coli 8178 tonB and fepB mutant is not attributed to iron acquisition but rather a defect in microcin-H47 production.

by:

[L294] *Production of active microcin H47 molecules involves a multistage process including the synthesis of the siderophore carrier, the microcin H47 precursor and their maturation (Rodríguez et al., 1999; Vassiliadis et al., 2010). Using our plasmid reporter tool, we discovered that the expression of the microcin H47 synthesis cluster was comparable among E. coli 8178 WT, tonB and fepB strains under iron-limited condition (S4D Fig). This indicated that disruption of tonB and fepB may impact siderophore production and/or microcin maturation instead. Together, these data suggest that the attenuated S. Tm competition capacity of the E. coli 8178 tonB and fepB mutant is not attributed to iron acquisition but rather a defect in the formation of mature microcin H47 molecules.*

We additionally revised the discussion and changed:

Mechanistically, due to their role in the uptake of siderophore, one could hypothesize that deletion of tonB or fepB leads to an ineffective siderophore intake, thereby lowering the intracellular pool of salmochelin which ultimately hinder microcin H47 attachment and killing.

by:

[L367] *Mechanistically, disruption of tonB or fepB in E. coli 8178 did not directly impact the expression of the microcin H47 synthesis gene cluster (S4D Fig), suggesting a potential effect at the level of siderophore synthesis and/or the microcin H47 maturation step instead.*

As for identifying the specific siderophore synthesis/maturation step impacted by TonB and understanding the mechanistic details behind it, we believe that addressing these questions would require a substantial amount of work that fall beyond the reviewing timeframe and the scope of the manuscript.

References:

- Gómez-Santos, N., Glatter, T., Koebnik, R., Świątek-Połatyńska, M. A., & Søgaard-Andersen, L. (2019). A TonB-dependent transporter is required for secretion of protease PopC across the bacterial outer membrane. *Nature communications*, *10*(1), 1360.
- Jurėnas, D., Fraikin, N., Goormaghtigh, F., & Van Melderen, L. (2022). Biology and evolution of bacterial toxin–antitoxin systems. *Nature Reviews Microbiology*, *20*(6), 335-350.
- Salamov, V. S. A., & Solovyevand, A. (2011). Automatic annotation of microbial genomes and metagenomic sequences. *Metagenomics and its applications in agriculture, biomedicine and environmental studies*, 61-78.

— — — — —

Reviewer#3:

In this manuscript, Cherrak et al. demonstrate that the murine commensal E. coli 8178 limits Salmonella expansion in the murine gut during inflammation. Specifically, the authors find that E. coli 8178 relies on the production of microcin-H47, a member of the antimicrobial microcin toxin family that when fused to salmochelin, limits the growth of Salmonella by acting as a trojan horse. Overall, the work presents compelling mechanistic data, showing how a murine gut commensal limits the growth of an invading pathogen. However, there are several concerns outlined below that should be addressed.

Response: We thank referee#3 for investing time in carefully reviewing our manuscript and appreciate the enthusiasm expressed. We hope our detailed point-by-point responses will be sufficient to strengthen our claim and support publication of our work.

1) The connection between inflammation and microcin-H47 production seems to be missing. While the authors cite seminal studies that demonstrate how "inflammation-mediated iron deprivation triggers siderophores utilization" by Salmonella, this study does not specifically test whether inflammation serves as a cue for microcin-H47 production. Experiments that demonstrate that inflammation regulates the production of microcin-H47 production by E. coli are needed.

Response: We thank referee#3 for bringing this to our attention. In the initial section of the manuscript, we described an inflammation-driven competition visible between *E. coli* 8178 and *S*. Tm (Figure 1D). While investigating the underlying mechanism, we found that *E. coli* 8178 relies predominantly on microcin H47 to outcompete *S*. Tm in the inflamed gut. This led us to speculate that inflammation triggers the production of microcin H47. To validate this hypothesis and considering the technical challenges associated with detecting microcin H47 molecules in an *in vivo* context, we opted for a genetic approach. Specifically, we designed an *E. coli* 8178 strain lacking the microcin H47 precursor synthesis gene (*mchB*) and the microcin H47 immunity (*mchI*), rendering it susceptible to microcin H47-dependent killing by another strain. We expected that the *mchB*/*mchI* strain will be outcompeted - in a microcin H47-dependent fashion - by the WT *E. coli* 8178 strain, which we demonstrated in Figure 4D. Indeed, the fitness of the *mchB*/*mchI* was highly diminished by the WT strain. This was achieved in the inflamed gut (Figure 4D, white dots) and in a microcin H47-dependent fashion (Figure 4D, green dots). Notably, the *mchB*/*mchI* fitness appears unaffected in presence of the WT strain under non inflamed conditions (Figure 4D, grey dots). From that, we can confidently claim that inflammation triggers microcin H47-dependent killing.

To further demonstrate this aspect, we have now designed a transcriptional reporter plasmid by fusing the promoter of the microcin H47 synthesis gene cluster to a nano-luciferase. Upon inoculating mice with the *E. coli* 8178 bearing the reporter plasmid (p-*PmchI-luc),* we observed a strong luciferase signal at 96 h post-infection under inflamed conditions, which was markedly reduced in non-inflamed animals. Based on this and our previous data, we can assert that microcin H47 synthesis is triggered by inflammation.

We have added this data in Figure 4C, Supplementary Fig 4B and revised the text accordingly. Specifically, we have replaced:

To further demonstrate E. coli 8178's ability to produce microcin H47 in the inflamed gut, we designed an E. coli 8178 mchI/mchB double mutant which lacks the gene responsible for microcin immunity and synthesis, making it susceptible to microcin-dependent killing by the WT strain. Triple infection experiments with S. Tm along with the WT and mchI/mchB E. coli 8178 strains resulted in the attenuation of the microcin-sensitive mutant in a microcin H47- and inflammation-dependent manner (Figure 4C).

by:

[L255] *To demonstrate E. coli 8178's ability to produce microcin H47 in the inflamed gut, we created a plasmid-based reporter (p-PmchI-luc) expressing a luciferase gene (Hall et al., 2012) under the control of the microcin*

H47 synthesis gene cluster promoter. Inoculation of S. Tm infected mice with E. coli 8178 bearing the p-PmchI-luc reporter resulted in a strong luciferase signal visible at 96 h post-infection, which was drastically reduced in animals experiencing no severe intestinal inflammation (Fig 4C, S4B). To further prove that inflammation acts as a cue to trigger microcin H47-dependent competition, and considering the difficulties associated with detecting microcin H47 from in vivo samples, we designed an E. coli 8178 mchI/mchB double mutant. This strain lacks the gene responsible for microcin immunity and synthesis, making it susceptible to microcin-dependent killing by the WT strain. Triple infection experiments with S. Tm along with the WT and mchI/mchB E. coli 8178 strains resulted in the attenuation of the microcin-sensitive mutant in a microcin H47- and inflammation-dependent manner (Fig 4D). Finally, in trans expression of the mchI immunity gene in S. Tm increased the pathogen's survival against E. coli 8178 (Fig 4E; S4C).

2) Lipocalin-2 is used as a general readout of inflammation in the murine gut. Other measures of inflammation with more context are recommended, such as proinflammatory cytokines. Moreover, Fig 1C and Supplementary Fig 1B have different cutoffs for what is considered inflamed vs normal (102 in 1C vs 103 in S1B). The same cutoff should be used in both graphs.

Response: We thank referee#3 for pointing out the different cutoffs in Figure 1C and Supplementary Figure 1B, which has now been normalized to 10^2 to align with previous published data (Nguyen et al., 2020; Diard et al., 2021).

Lipocalin-2 serves as a widely accepted marker for gut inflammation, utilized not only by our research group but also many other laboratories studying intestinal diseases (Chassaing et al., 2012; Moschen et al., 2017; Herp et al., 2019; Nguyen et al., 2020, Ruddle et al., 2023). Besides its widespread use, recent findings from our laboratories have shown a clear correlation between the levels of intestinal lipocalin-2 and the expression of proinflammatory genes (Cxcl9, Cxcl10, Interferon gamma, Il1β: Gül et al., 2023). Based on that, and the body of literature on our *S*. Tm SL1344 WT and *spi*-1/*spi*-2 mutant (Stecher et al., 2007), we believe that the level of lipocalin-2 depicted on Fig 1C and Supplementary Fig 1B accurately reflects the degree of intestinal inflammation observed in our mice. To further support this conclusion, we have conducted histopathology scoring of the caecal tissue from mice infected with the *S*. Tm WT or *spi*-1/*spi*-2 mutant at 96 h post-infection. In contrast to cytokines measurement which provides indirect information about relevant responses that may alter the gut-luminal milieu, the enteropathy scoring provides more direct information. As expected, samples from *S*. Tm-infected mice displayed elongated crypts, epithelium gaps, sub-mucosal edema and polymorphonuclear leukocytes infiltration, indicative of a severe intestinal inflammation which nicely correlates with the high level of lipocalin-2 in these animals. In contrast, mice infected with the S. Tm *spi*-1/*spi*-2 displayed attenuated intestinal injuries characterized by lower histopathological scores and lipocalin-2 levels. We conclude that lipocalin-2 levels depicted in the manuscript accurately mirrors the extent of intestinal inflammation and therefore provide reasonable evidence to support our conclusions regarding the role of inflammation in *E. coli* 8178-mediated *S*. Tm competition.

The histopathology scores and tissue sections are depicted in Supplementary Fig 1D.

3) Are growth rates of 8178 mutants and Salmonella strains expressing genes in trans altered? Are dynamics of colonization different?

To ensure consistent colonization kinetics among all *E. coli* 8178 metabolic mutants designed in our study, we have analysed their *in vivo* fitness under both inflamed and non-inflamed condition at 24 h post-infection. As illustrated in Supplementary Fig 2A and Supplementary Fig 3C, not all of the *E. coli* 8178 metabolic mutants tested displayed similar growth kinetics to the WT strain in the mouse gut. For instance, the *fhuE* mutant exhibited a 10-fold decrease in load compared to the WT strain under non-inflamed conditions. In contrast, the *E. coli* 8178 *iroN* and *chuA* strains appeared to possess a fitness advantage over the WT in these conditions. Given that we are specifically targeting genes known to impact bacterial growth in the gut, we anticipated that some of our mutants would experience growth changes. This is why we focused instead on *E. coli* 8178 mutant strains that displayed a distinct competitive index across the two conditions tested (inflamed vs non-inflamed), with a primary emphasis on strains that demonstrated a greater disadvantage during inflammation compared to non-inflamed conditions.

Regarding the other *E. coli* 8178 strains tested (i.e. toxin deficient…), we initially believed that indicating their loads at 96 h post-infection was sufficient to support our findings. However, we acknowledge the referee's point that differences in the initial gut colonization may contribute to the observed phenotypes. Consequently, we have now updated Supplementary Fig 2B, Supplementary Fig 3A, D, E, and Supplementary Fig 4A with the load of each *E. coli* 8178 mutant at 24 h post-infection. These data indicate that none of the strains tested displayed an initial colonization defect.

As for the strains expressing genes *in trans*, we would like to bring to the referee's attention that we did not use any *E. coli* 8178 strain expressing gene *in trans* in the previous version of the manuscript. However, with the addition of the *E. coli* 8178 reporter strain, we have included the bacterial loads at 24 h and 96 h post-infection, as shown in Supplementary Fig 4B.

Finally, we have analysed the colonization properties of the *S*. Tm strain bearing the p-*mchI* plasmid allowing the constitutive expression of the microcin immunity protein (Fig 4E) and compared it to the WT strain. In absence of *E. coli* 8178, the S. Tm + p-*mchI* strain exhibits no change in fitness (C.I: ~1) at 24 h and 96 h post-infection (Supplementary Fig 4C). This confirmed that its increased survival observed in Fig 4E results solely from the microcin immunity conferring protection against microcin H47-dependent killing.

4) The conclusion from Figure 4 that "...the attenuated S. Tm competition capacity of the E. coli 8178 tonB and fepB mutant is not attributed to iron acquisition but rather a defect in microcin-H47 production" should be strengthened through additional experimentation. The authors should directly and quantitatively measure microcin-H47 production, such as through an ELISA, in the E. coli 8178 tonB and fepB mutants to strengthen the conclusion that the strains are deficient in microcin-H47 synthesis.

Response: We thank referee#3 for pinpointing the lack of evidence supporting the statement: "the attenuated S. Tm competition capacity of the *E. coli* 8178 *tonB* and *fepB* mutant is not attributed to iron acquisition but rather a defect in microcin-H47 production" sentence. In response also to referee's 1 request, we have now further clarified this point with additional data. Specifically, we demonstrated that an *E. coli* 8178 strain unable to synthesize microcin H47 and uptake salmochelin (*mchB*/*iroN*) phenocopies the microcin H47-deficient strain (*mchB*). This data, which is now incorporated in the updated version of the manuscript (Fig 4B), indicates that salmochelin uptake has no contribution on the *E. coli* 8178 – *S*. Tm competition visible under our conditions.

Regarding the impaired microcin H47 production in the *E. coli* 8178 *tonB* and *fepB* mutant background, we agree that biochemical evidence could nicely complement our genetic approaches. However, it is important to note that, to our knowledge, there are no ELISA kits currently available that are specifically designed from the detection of microcin-H47, particularly from *in vivo* samples. Isolation/quantification of microcin H47 relies instead on mass spectrometry approaches from defined media (Vassiliadis et al., 2010; Henderson et al., 2009; Behnsen et al., 2021), which differs from complex gut intestinal samples posing additional challenges. Confronted with this limitation, we have employed an alternative approach to demonstrate the impairment of microcin H47 synthesis in the *E. coli* 8178 *tonB* and *fepB* mutants. Previously, we showed that the deletion of the microcin H47 synthesis (*mchB*) and immunity (*mchI*) genes in *E. coli* 8178 (*mchB*/*mchI* double mutant) makes the strains susceptible to microcin H47-dependent killing (Fig 4D). Building upon this, we have designed triple *tonB*/*mchB*/*mchI* and *fepB*/*mchB*/*mchI* mutant strains to serve as proxies for the production of microcin H47 in the respective *E. coli* 8178 *tonB* and *fepB* mutants. Notably, the microcin H47-sensistive *E. coli* 8178 *mchB*/*mchI*/*tonB* strain was not outcompeted by the *E. coli* 8178 *tonB* mutant. Similar observations were made for the *mchB*/*mchI*/*fepB* strain in comparison to the *E. coli* 8178 *fepB* mutant. Based on this evidence and in line with our *in vitro* data, we concluded that *tonB* and *fepB* disruption leads to a defect in microcin H47-dependent killing. We have incorporated these additional data in Fig 4G and updated the result section by replacing:

In comparison to the WT strain, the intracellular fraction of the E. coli 8178 tonB and fepB mutant did not inhibit E. coli K-12 growth, indicating a defect in the synthesis of active microcin H47 in these strains. Together, these data suggest that the attenuated S. Tm competition capacity of the E. coli 8178 tonB and fepB mutant is not attributed to iron acquisition but rather a defect in microcin-H47 production.

by:

[L287] *In comparison to the WT strain, the intracellular fraction of the E. coli 8178 tonB and fepB mutant did not inhibit E. coli K-12 growth, indicating a defect in the synthesis of active microcin H47 in these strains. To confirm the impaired microcin H47-dependent killing upon disruption of tonB and fepB from an in vivo perspective, we engineered an E. coli 8178 tonB and fepB mutant susceptible to microcin H47 (E. coli 8178 tonB/mchB/mchI and fepB/mchB/mchI) and analysed their fitness under inflamed conditions. In line with our in vitro data, the E. coli 8178 tonB and fepB mutant failed to outcompete the microcin H47-sensitive tonB and fepB strains (Fig 4G).*

Lastly, we would like to draw the referee's attention that we measured the expression of the microcin H47 synthesis gene in the *E. coli* 8178 *tonB* and *fepB* background using our newly developed transcriptional reporter plasmid (p-*PmchI-luc)*. Under *in vitro* conditions, we found that the *E. coli* WT, *tonB* and *fepB* exhibited comparable levels of microcin H47 synthesis gene expression (Supplementary Fig 4D). This suggests that disruption of *tonB* or *fepB* impairs the formation of microcin H47 molecules by impacting the siderophore synthesis and/or attachment. This observation, coupled to the technical challenges posed by the detection of microcin H47 *in vivo*, forced us to use an alternative genetic approach to further strengthen our claim.

5) Inclusion of simple models of in vitro competition would strengthen the study. As it is postulated that inflammation leads to iron deprivation that causes Salmonella to use siderophores, it would be an important "proof-of-concept" experiment to see E. coli 8178 outcompete Salmonella in vitro under iron deprivation conditions in culture to isolate this mechanism.

Response: This is an extremely relevant question, and we thank referee#3 for bringing this up. We have discovered that *S*. Tm-elicited inflammation triggers pathogen elimination by the commensal *E. coli* 8178 through a mechanism relying on microcin H47. Given that microcin H47 comprises a siderophore backbone that aids in delivering the toxic molecule to siderophore-utilizing competitors, we expected that iron deprivation (which commonly occurs during *S*. Tm-elicited gut inflammation: Deriu et al., 2013) would act as a cue triggering *E. coli* 8178-mediated *S*. Tm elimination. Unfortunately, all our attempts to replicate this phenomenon in an *in vitro* set-up have failed (even under iron-limited conditions). Nonetheless, we could easily recapitulate microcin H47-dependent killing using a different target strain (*E. coli* MG1655). This led us to conclude that, unlike *E. coli* MG1655, additional factors present during intestinal inflammation may be necessary to induce *S*. Tm growth inhibition by microcin H47. As suggested by the referee, we believe that recreating an *in vitro* inflammation context would be extremely useful in recapitulating our *in vivo* observations. However, given the complexity of the inflammation process, it remains extremely challenging to achieve it.

6) In the molecular biology section of the methods, a microcin expression reporter is mentioned that is not used at all in the text.

Response: We have now updated this section and the tables to mention the microcin H47-reporter plasmid designed in the study.

7) In lines 110 and 111, the authors note: "Apart from the early stages of infection (24hpi), the presence of E. coli 8178 did not impact the onset of S. Tm-elicited inflammation." The authors should address the slight but statistically significant decrease in S. Tm-elicited inflammation in the presence of E. coli 8178 at 24 hpi. Is it biologically meaningful?

Response: We thank the reviewer for highlighting the lipocalin-2 differences observed at 24 h post-infection. Despite reducing *S*. Tm loads by approximately 1000-fold, the presence of *E. coli* 8178 did not diminish the level of intestinal inflammation at 96 h post-infection. As microcin H47-dependent killing is triggered by inflammation, S. Tm eliminations occurs only after inflammation is already established.

In contrast, we observed a change in the lipocalin-2 levels at 24 h post-infection between *S*. Tm mono-infected and *S*. Tm + *E. coli* co-infected mice (Fig 1C). The attenuation of intestinal inflammation by *E. coli* 8178 at that stage correlates nicely with the reduction of *S*. Tm loads observed at 24 h post-infection (Fig 1B). As we are currently investigating this phenotype further, we believe that this difference most likely results from a second type of competition, which appears to be microcin H47-independent and instead relies on metabolic exploitation.

Given our limited understanding of this phenomenon at that stage and the primary focus of the study which is centred around the competition observed at 96h post-infection, we chose not to further interpret this phenomenon.

Meanwhile, we have further highlighted the difference observed at 24 h post-infection. Specifically, we have replaced:

In comparison, coinfection with a 1:1 ratio of S. Tm and E. coli 8178 led to equivalently high gut luminal loads after 48h of infection which drastically declined (1.000- to 10.000-fold) 72 to 96 h post-infection (Figure 1B, Supplementary Figure 1A). We concluded that similarly to C57BL/6 mice (Stecher et al., 2012), E. coli 8178 can attenuate S. Tm growth in antibiotic-pretreated 129SvEv/Tac animals.

by:

[L103] *Besides the slight decrease (~ 10-fold) in S. Tm loads visible at the early stage of infection (24 h p.i), co-infection with a 1:1 ratio of S. Tm and E. coli 8178 led to equivalently high gut pathogen luminal loads after 48 h of infection (**Fig 1B**, **S1A**). This load displayed a more striking decline (1.000- to 10.000-fold) 72 to 96 h post-infection (**Fig 1B**, **S1A**) which aligned with previous observation in C57BL/6 mice (Stecher et al., 2012) and prompted us to investigate the underlying mechanisms*.

8) In Figure 2B, it is intriguing that is a statistically significant difference in the competitive index for the Δfrdabcd E. coli mutant in the presence of WT Salmonella compared to the SPI-1/SPI-2 mutant of Salmonella. This should be more specifically addressed.

Fumarate respiration, facilitated by the *frdABCD* cluster, has been demonstrated to be crucial for the anaerobic growth of several bacterial strains, including *E. coli* (Jones et al., 2011) and *S*. Tm (Nguyen et al., 2020) *in vivo*. Consistent with these findings, we demonstrated that *E. coli* 8178 relies on the *frdABCD* cluster to efficiently grow in our mouse model (C.I < 1; Fig 2B). This holds true in the presence of the WT or *spi*-1/*spi*-2 avirulent *S*. Tm strains (Fig 2B). Notably, the growth defect of a *E. coli* 8178 *frdABCD* mutant is more pronounced in a non-inflamed condition (C.I: ~0.01) compared to an inflamed gut (C.I: ~0.1). We believe that the differential requirement for fumarate respiration across these two conditions arises from the presence of additional electron acceptors released during *S*. Tm elicited inflammation. In contrast to a non-inflamed context where *E. coli* 8178 appears to depend significantly on fumarate respiration, the presence of additional electron acceptors liberated during gut inflammation (*i.e* tetrathionate: Winter et al., 2010; nitrate: Winter et al., 2013; oxygen: Spiga et al., 2017) seems to alleviate fumarate-respiration dependent growth of *E. coli* 8178 under this condition. As a result, the growth defect of the *E. coli* 8178 *frdABCD* mutant is less pronounced in the inflamed gut.

We have clarified that aspect on the text and changed:

Notably, E. coli 8178 moaA, tonB and cydAB mutants exhibited a competitive disadvantage exclusively in presence of the WT S. Tm strain, while no fitness defect was observed in presence of the S. Tm spi-1/spi-2 mutant ***(Figure 2B)***

by:

[L156] *In line with earlier work indicating the release of electron acceptors (different than fumarate) during S. Tm-elicited inflammation (Winter et al., 2010; Winter et al., 2013a; Spiga et al., 2017), an E. coli 8178 incapable of fumarate respiration (ΔfrdABCD) exhibited a competitive disadvantage that was less pronounced in the inflamed than in the non-inflamed gut (**Fig 2B**). Conversely, the fitness of the E. coli 8178 moaA, tonB and cydAB mutant was exclusively attenuated in the presence of the WT S. Tm strain, while remaining neutral in mice co-infected with the S. Tm spi-1/spi-2 mutant (**Fig 2B**).*

Additionally, we would like to bring to the referee's attention that, considering the *E. coli* 8178-mediated competition observed under inflamed conditions, we specifically screened for metabolic mutants that displayed (1) a neutral fitness in presence of the *spi*-1/*spi*-2 mutant (C.I: 1) and (2) a growth disadvantage in presence of the WT S. Tm (C. I < 1). As the *E. coli* 8178 *frdABCD* mutant did not meet these criteria, we excluded it from further analysis.

9) For Figure 2A, the labels of the axes on the graph should be enlarged for improved readability.

Response: We have increased the size of the labels.

10) Table S3 is incomplete. Primers for construction of deletion strains are listed, but other primers such as those used for generating PmchI need to be included as well.

Response: We have now updated the table S3 to include the primers used for designing the constitutively expressing microcin immunity and microcin H47 reporter plasmids.

References:

- Behnsen, J., Zhi, H., Aron, A. T., Subramanian, V., Santus, W., Lee, M. H., ... & Raffatellu, M. (2021). Siderophore-mediated zinc acquisition enhances enterobacterial colonization of the inflamed gut. *Nature communications*, *12*(1), 7016.
- Chassaing, B., Srinivasan, G., Delgado, M. A., Young, A. N., Gewirtz, A. T., & Vijay-Kumar, M. (2012). Fecal lipocalin 2, a sensitive and broadly dynamic non-invasive biomarker for intestinal inflammation.
- Diard, M., Bakkeren, E., Lentsch, V., Rocker, A., Bekele, N. A., Hoces, D., ... & Slack, E. (2021). A rationally designed oral vaccine induces immunoglobulin A in the murine gut that directs the evolution of attenuated Salmonella variants. *Nature microbiology*, *6*(7), 830-841.
- Gül, E., Bakkeren, E., Salazar, G., Steiger, Y., Abi Younes, A., Clerc, M., ... & Hardt, W. D. (2023). The microbiota conditions a gut milieu that selects for wild-type Salmonella Typhimurium virulence. *PLoS biology*, *21*(8), e3002253.
- Henderson, J. P., Crowley, J. R., Pinkner, J. S., Walker, J. N., Tsukayama, P., Stamm, W. E., ... & Hultgren, S. J. (2009). Quantitative metabolomics reveals an epigenetic blueprint for iron acquisition in uropathogenic Escherichia coli. *PLoS pathogens*, *5*(2), e1000305.
- Herp, S., Brugiroux, S., Garzetti, D., Ring, D., Jochum, L. M., Beutler, M., ... & Stecher, B. (2019). Mucispirillum schaedleri antagonizes Salmonella virulence to protect mice against colitis. *Cell host & microbe*, *25*(5), 681-694.
- Jones, S. A., Gibson, T., Maltby, R. C., Chowdhury, F. Z., Stewart, V., Cohen, P. S., & Conway, T. (2011). Anaerobic respiration of Escherichia coli in the mouse intestine. *Infection and immunity*, *79*(10), 4218-4226.
- Moschen, A. R., Adolph, T. E., Gerner, R. R., Wieser, V., & Tilg, H. (2017). Lipocalin-2: a master mediator of intestinal and metabolic inflammation. *Trends in Endocrinology & Metabolism*, *28*(5), 388-397.
- Nguyen, B. D., Cuenca, M., Hartl, J., Gül, E., Bauer, R., Meile, S., ... & Hardt, W. D. (2020). Import of aspartate and malate by DcuABC drives H2/fumarate respiration to promote initial Salmonella gut-lumen colonization in mice. *Cell host & microbe*, *27*(6), 922-936.
- Ruddle, S. J., Massis, L. M., Cutter, A. C., & Monack, D. M. (2023). Salmonella-liberated dietary L-arabinose promotes expansion in superspreaders. *Cell Host & Microbe*, *31*(3), 405-417.
- Spiga, L., Winter, M. G., de Carvalho, T. F., Zhu, W., Hughes, E. R., Gillis, C. C., ... & Winter, S. E. (2017). An oxidative central metabolism enables Salmonella to utilize microbiota-derived succinate. *Cell Host & Microbe*, *22*(3), 291-301.
- Vassiliadis, G., Destoumieux-Garzón, D., Lombard, C., Rebuffat, S., & Peduzzi, J. (2010). Isolation and characterization of two members of the siderophore-microcin family, microcins M and H47. *Antimicrobial agents and chemotherapy*, *54*(1), 288-297.
- Winter, S. E., Thiennimitr, P., Winter, M. G., Butler, B. P., Huseby, D. L., Crawford, R. W., ... & Bäumler, A. J. (2010). Gut inflammation provides a respiratory electron acceptor for Salmonella. *Nature*, *467*(7314), 426-429.
- Winter, S. E., Winter, M. G., Xavier, M. N., Thiennimitr, P., Poon, V., Keestra, A. M., ... & Bäumler, A. J. (2013). Host-derived nitrate boosts growth of E. coli in the inflamed gut. *science*, *339*(6120), 708-711.

— — — — —

Reviewer #4:

In this manuscript by Cherrak et al the authors examine the mechanism by which a commensal E. coli strain outcompetes Salmonella Typhimurium in mice. The competition coincides with and is dependent on inflammation triggered by the Salmonella Spi1 and Spi2 T3SS. They examine a number of candidate genes in E. coli and find that tonB, which has pleiotropic functions, is necessary for E. coli to outcompete Salmonella. They further examine tonB-dependent pathways and find that some iron uptake siderophore-related genes are necessary. Contrary to previous assumptions, the salmochelin siderophore-related genes are not needed purely for iron uptake, but also as part of a bacterial killing mechanism wherein a microcin is conjugated to salmochelin. This is a nice addition to the colonization resistance literature and will be of interest to PLOS Biology readers. However, the study has limitations as it compares with physiological colonization resistance. In addition, there are several points that need to addressed by the authors.

Response: We would like to express our gratitude to referee#4 for dedicating time reviewing our manuscript. We hope the revised version and subsequent point-by-point answer will be sufficient to strengthen our claims and support publication of our work.

1. The experimental approach used by the authors relies on co-infection of Salmonella and commensal E. coli. However, this is not the normal context in which symbiotic bacteria inhibit Salmonella colonization. Furthermore, the levels of colonization of symbiotic E. coli are far lower under normal conditions that in the experimental design used by the authors. The authors may be overstating their conclusions in the context of normal Salmonella infection. Thesa authors need to address this point.

Response: We acknowledge the observation made by referee#4 regarding our infection procedure. Indeed, as *E. coli* 8178 is provided concomitantly, and is not present prior *S*. Tm inoculation, this setup does not precisely replicate the typical inhibition by symbiotic bacteria. To better simulate this scenario, we inoculated streptomycin-pretreated mice with *E. coli* 8178 prior to *S*. Tm infection. Under these conditions, *S*. Tm was outcompeted in a similar fashion as in co-infected mice. This indicated that the outcome of the *S*. Tm – *E. coli* 8178 competition remains consistent regardless of the setup used. We opted for co-infection experiments throughout the entire manuscript as it provided us a better controlled, replicable, and manipulable framework to confidently interpret our findings.

We have placed this additional data in Supplementary Fig 1B, C and added in the results section:

[L108] *In that perspective and although S. Tm was similarly outcompeted in E. coli 8178-preinoculated animals (S1B, 1C Fig), we opted for a coinfection experimental setup.*

We also agree that the *E. coli* loads (10^9 – 10^{10} c.f.u/g feces) observed in our mouse model may not precisely reflect the loads under physiological conditions. Indeed, *E. coli* and Enterobacteriaceae in general, are frequently present in relatively low abundance, but this can vary greatly depending on the context. For example, colonization level of *E. coli* was shown to exhibit inter-individual variations, ranging up to 10^9 c.f.u/g feces (Tenaillon et al., 2010; Wotzka et al., 2018). Moreover, inflammation is associated with a bloom of *Enterobacteriaceae*, resulting in their predominance in the intestinal gut (Reviewed in Zeng et al., 2016). Based on this evidence, we believe that the load of *E. coli* 8178 reported in our mouse model, although not entirely physiological, may mirror the load of *E. coli* strains in individuals under certain circumstances.

We have further highlighted this limitation in the Discussion section and replaced:

The preference for such diffusible toxins may be attributed to their long-range effect, which particularly benefit a population experiencing high loads, as E. coli 8178 encounters during gut inflammation (Supplementary Fig 1A; Stecher et al., 2012; Booth et al., 2023).

by:

[L324] *The preference for such diffusible toxins may be attributed to their long-range effect, which particularly benefit a population experiencing high loads, as E. coli 8178 encounters during gut inflammation (S1A Fig) (Stecher et al., 2012; Booth et al., 2023). However, it is important to note that E. coli and more generally members of the Enterobacteriaceae family are underrepresented in the healthy gut (Lozupone et al., 2012), which consequently limits the average toxin concentration at the luminal side. It remains yet to be determined whether diffusible molecules such as microcins can still provide benefits under these circumstances.*

2. Classical colonization resistance provided by the microbiota was originally defined in the 1950' early during infection (first 24 hrs) in which the microbiota reduces Salmonella colonization over 5-logs. in mice. The authors need to mention this point to avoid confusion to the readers. The evidence is that this robust and early colonization resistance in mice and birds is mediated by obligate anaerobes particularly Clostridia. This point should be mentioned in the Introduction. In addition, the title and abstract should incorporate the notion that the protective effect of E. coli observed by the authors was late and correlated with inflammation.

Response: We thank referee#4 for highlighting missing details in our title/abstract/introduction. As requested, we have changed the introduction section and are now referring to historical works on colonization resistance. Specifically, we have replaced:

By alleviating gut colonization resistance, these approaches allow efficient gut infections by S. Tm (Barthel et al., 2003; Brugiroux et al., 2016). Colonization resistance refers to the ability of an unperturbed gut microbiota to act as a natural protective barrier restricting the growth of incoming pathogens (Buffie & Pamer 2013; Caballero-Flores et al., 2023).

by:

[L41] *By alleviating gut colonization resistance, these approaches allow efficient gut infections by S. Tm (Barthel et al., 2003; Brugiroux et al., 2016). Colonization resistance, identified in the 1950s after observing a significant increase in intestinal S. Tm loads upon antibiotic pretreatment (Bonhoff et al., 1954), refers to the ability of an unperturbed gut microbiota to act as a natural protective barrier restricting the growth of incoming pathogens (Buffie & Pamer 2013; Caballero-Flores et al., 2023).*

Related to protective effect of *Clostridia* we agree with the referee that members of the *Clostridia* class play a predominant role in limiting the invasion of enteropathogens, which we are now referring in the introduction as:

[L52] *In a similar fashion, depletion of Clostridia was shown to increase luminal oxygenation favouriting S. Tm bloom (Rivera-Chávez et al., 2016), while the human probiotic E. coli Nissle 1917 strain (Mutaflor®) has demonstrated an efficient ability to outcompete S. Tm via oxygen depletion (Litvak et al., 2019; Velazquez et al., 2019).*

We also recognize that colonization resistance was historically associated with an earlier growth inhibition (first 24h). To remove any confusion, we have now uncoupled the phenotype studied with colonization resistance and instead refer to bacterial antagonistic relationships. We have replaced:

By identifying the E. coli 8178's genetic determinants mediating S. Tm competition, our work not only provides a better mechanistic understanding of the protective function displayed by members of the gut microbiota but also further expand the general contribution of microcins in colonization resistance.

by:

[L26] *By identifying the genetic determinants mediating S. Tm competition, our work not only provides a better mechanistic understanding of the protective function displayed by members of the gut microbiota but also further expand the general contribution of microcins in bacterial antagonistic relationships.*

and:

In this study, we investigated how commensal E. coli mediate colonization resistance against Salmonella enterica serovar Typhimurium (S. Tm).

by:

[L18] *In this study, we investigated how commensal E. coli can outcompete Salmonella enterica serovar Typhimurium (S. Tm).*

and:

Colonization resistance conferred by the gut microbiota can be traced to specific commensal strains exhibiting direct or indirect protective functions.

by:

[L14] *The microbiota can limit gut-luminal pathogen growth at different stages of infection. This can be traced to specific commensal strains exhibiting direct or indirect protective functions.*

Lastly, we have modified the title and abstract to include the contribution of inflammation in our phenotype. Specifically, we have replaced:

A commensal E. coli strain limits Salmonella gut invasion by producing toxin-bound siderophores in a tonB-dependent manner.

by:

[Title] *A commensal E. coli strain limits Salmonella gut invasion during inflammation by producing toxin-bound siderophores in a tonB-dependent manner.*

and:

Using a salmonellosis mouse model coupled with bacterial genetic approaches, we found that the commensal E. coli 8178 strain relies on a trojan horse trap strategy to limit S. Tm expansion in the mammalian gut.

by:

[L19] *Using a salmonellosis mouse model coupled with bacterial genetic approaches, we found that the commensal E. coli 8178 strain relies on a trojan horse trap strategy to limit S. Tm expansion in the inflamed gut.*

3. 22 - "we demonstrated that inflammation triggers the expression of the E. coli 8178 antimicrobial microcin…" Strictly speaking, microcin expression wasn't evaluated. It may be induced by inflammation in vivo but also in vitro by iron deprivation, apparently (Fig. 4E). This should be reworded for accuracy.

Response: We thank referee#4 for highlighting the lack of direct evidence for the inflammation-driven microcin H47 production in *E. coli* 8178. We have now revised this aspect by assessing the expression level of the microcin H47 synthesis gene cluster, *in vivo*, under both inflamed and non-inflamed condition. In that perspective, we have engineered a transcriptional reporter plasmid by fusing the promoter of the microcin H47 synthesis gene cluster to a nano-luciferase (*p-PmchI-luc*). Upon inoculating mice with an *E. coli* 8178 strain bearing the reporter plasmid, we observed a strong luciferase signal at 96 h post-infection under inflamed conditions, which was markedly reduced in non-inflamed animals. Based on this, we can assert that microcin H47 synthesis is triggered by inflammation.

We have added this data in Figure 4C and revised the text accordingly. Specifically, we have replaced:

*To further demonstrate E. coli 8178's ability to produce microcin H47 in the inflamed gut, we designed an E. coli 8178 mchI/mchB double mutant which lacks the gene responsible for microcin immunity and synthesis, making it susceptible to microcin-dependent killing by the WT strain. Triple infection experiments with S. Tm along with the WT and mchI/mchB E. coli 8178 strains resulted in the attenuation of the microcin-sensitive mutant in a microcin H47- and inflammation-dependent manner (**Figure 4C**).*

by:

[L255] *To demonstrate E. coli 8178's ability to produce microcin H47 in the inflamed gut, we created a plasmid-based reporter (p-PmchI-luc) expressing a luciferase gene (Hall et al., 2012) under the control of the microcin H47 synthesis gene cluster promoter. Inoculation of S. Tm infected mice with E. coli 8178 bearing the p-PmchI-*

luc reporter resulted in a strong luciferase signal visible at 96 h post-infection, which was drastically reduced in animals experiencing no severe intestinal inflammation (Fig 4C, S4B). To further prove that inflammation acts as a cue to trigger microcin H47-dependent competition, and considering the difficulties associated with detecting microcin H47 from in vivo samples, we designed an E. coli 8178 mchI/mchB double mutant. This strain lacks the gene responsible for microcin immunity and synthesis, making it susceptible to microcin-dependent killing by the WT strain. Triple infection experiments with S. Tm along with the WT and mchI/mchB E. coli 8178 strains resulted in the attenuation of the microcin-sensitive mutant in a microcin H47- and inflammation-dependent manner (Fig 4D). Finally, in trans expression of the mchI immunity gene in S. Tm increased the pathogen's survival against E. coli 8178 (Fig 4E; S4C).

Having demonstrated the inflammation act as a cue to induce microcin H47 production, we agree with referee#4 that our *in vitro* experiments might be potentially confusing as we demonstrated that microcin H47 can be triggered by iron deprivation. However, it is worth mentioning that iron deprivation is a phenomenon that frequently cooccurs upon inflammation (nutritional immunity: Hood & Skaar, 2012). This mechanism aims to restrict pathogen growth by limiting iron availability. Based on that, we believe iron deprivation during inflammation is the trigger for microcin H47-dependent killing, which we, and others (Sassonne-Corsi et al., 2016; Palmer et al., 2020), have demonstrated under iron-limited *in vitro* conditions. To clarify this, we have replaced:

For this aim, we established an in vitro killing assay and assessed the level of microcin H47 synthesis in different E. coli 8178 mutant backgrounds under iron-deprived condition.

by:

[L277] *For this aim, we established an in vitro killing assay and assessed the level of microcin H47 synthesis in different E. coli 8178 mutant backgrounds under iron-deprived condition, a cue cooccurring with intestinal inflammation and known to trigger microcin production (Sassonne-Corsi et al., 2016; Palmer et al., 2020).*

4. Fig. 3B - iroN is the salmochelin receptor and the mutant shows a phenotype in inflamed conditions here. It would be nice to comment on why this is so (does it also have a cryptic function in microcin synthesis?). Was it followed up in conditions as in Fig. 3C? Does it kill in the assay in Fig. 4E?

Response: This is a very relevant question, and we thank referee#4 for giving us the opportunity to elaborate on it.

During inflammation, the host restricts the availability of iron. To cope with host-mediated iron deprivation during inflammation, bacteria produce siderophores. Previous literature has shown that the salmochelin siderophore plays an important role during gut inflammation, aiding *S*. Tm in coping with iron limitation (Fischbach et al., 2006; Rafatellu et al., 2009). Consistently, we found that disruption of the salmochelin receptor *iroN*, whose primary function is iron-bound salmochelin uptake, leads to an attenuated growth in the inflamed gut, compared to non-inflamed conditions.

We have contextualized this aspect better in the manuscript by (1) mentioning the iron-restriction occurring during inflammation in the introduction and (2) explaining the growth fitness associated to *iroN* in Fig 3B. Specifically, we have replaced:

Besides oxygen intake, E. coli Nissle can outgrow S. Tm through iron acquisition.

by:

[L55] *Besides oxygen intake, E. coli Nissle can outgrow S. Tm through the acquisition of iron, a crucial metabolite whose availability is restricted during inflammation (Deriu et al., 2013).*

and:

Altogether, these observations indicated a potential role of catecholate intake in E. coli 8178-mediated S. Tm competition.

by:

[L207] *Altogether, these observations indicated catecholate siderophores, which play a role in mediating E. coli 8178's survival under the iron-limited inflamed, may potentially contribute to E. coli 8178-mediated S. Tm competition.*

Related to the absence of the *E. coli* 8178 *iroN* mutant in Fig 3C, we would like to bring to the referee's attention that the competitive index of this mutant in Fig 3B is ~10 under non-inflamed conditions and ~1 in presence of the WT *S.* Tm strain. This indicated that disruption of *iroN* in *E. coli* 8178 lead to a fitness advantage in the non-inflamed gut while remaining neutral in the inflamed gut. Considering that we were particularly screening for *E. coli* 8178 TBDR mutants exhibiting a growth defect (C.I <1) in the inflamed gut, and that the *iroN* mutant displayed a C.I of 1 under these conditions, we did not select it for further analysis.

We have now emphasized this aspect in the text, replacing:

To assess their competitiveness against S. Tm, the resulting E. coli 8178 mutants were individually barcoded, pooled, and their fitness assessed by qPCR in presence of either the S. Tm WT or spi-1/spi-2 strain (Figure 3B; Supplementary Figure 3C).

by:

[L197] *The resulting E. coli 8178 mutants were individually barcoded, pooled, and their fitness assessed by qPCR, with our primary focus on mutations that were (1) neutral when competing against the S. Tm spi-1/spi-2 strain and (2) attenuated in presence of the WT S. Tm at 96 h post-infection (Fig 3B; S3C).*

References:

- Fischbach, M. A., Lin, H., Zhou, L., Yu, Y., Abergel, R. J., Liu, D. R., ... & Smith, K. D. (2006). The pathogen-associated iroA gene cluster mediates bacterial evasion of lipocalin 2. *Proceedings of the National Academy of Sciences*, *103*(44), 16502-16507.
- Hood, M. I., & Skaar, E. P. (2012). Nutritional immunity: transition metals at the pathogen–host interface. *Nature Reviews Microbiology*, *10*(8), 525-537.
- Palmer, J. D., Mortzfeld, B. M., Piattelli, E., Silby, M. W., McCormick, B. A., & Bucci, V. (2020). Microcin H47: A Class IIb microcin with potent activity against multidrug resistant Enterobacteriaceae. *ACS Infectious Diseases*, *6*(4), 672-679.
- Raffatellu, M., George, M. D., Akiyama, Y., Hornsby, M. J., Nuccio, S. P., Paixao, T. A., ... & Bäumler, A. J. (2009). Lipocalin-2 resistance confers an advantage to Salmonella enterica serotype Typhimurium for growth and survival in the inflamed intestine. *Cell host & microbe*, *5*(5), 476-486.
- Sassone-Corsi, M., Nuccio, S. P., Liu, H., Hernandez, D., Vu, C. T., Takahashi, A. A., ... & Raffatellu, M. (2016). Microcins mediate competition among Enterobacteriaceae in the inflamed gut. *Nature*, *540*(7632), 280-283.
- Tenaillon, O., Skurnik, D., Picard, B., & Denamur, E. (2010). The population genetics of commensal Escherichia coli. *Nature reviews microbiology*, *8*(3), 207-217.
- Wotzka, S. Y., Kreuzer, M., Maier, L., Zünd, M., Schlumberger, M., Nguyen, B., ... & Misselwitz, B. (2018). Microbiota stability in healthy individuals after single-dose lactulose challenge—A randomized controlled study. *PLoS One*, *13*(10), e0206214.
- Zeng, M. Y., Inohara, N., & Nuñez, G. (2016). Mechanisms of inflammation-driven bacterial dysbiosis in the gut. Mucosal Immunol 10: 18–26.
